# Optimal Design of Wood/Rice Husk-Waste-Filled PLA Biocomposites Using Integrated CRITIC–MABAC-Based Decision-Making Algorithm

**DOI:** 10.3390/polym14132603

**Published:** 2022-06-27

**Authors:** Tej Singh, Punyasloka Pattnaik, Amit Aherwar, Lalit Ranakoti, Gábor Dogossy, László Lendvai

**Affiliations:** 1 Savaria Institute of Technology, Faculty of Informatics, Eötvös Loránd University, 9700 Szombathely, Hungary; sht@inf.elte.hu; 2 Department of Management Studies, Malaviya National Institute of Technology, Jaipur 302017, India; punyasloka2010@gmail.com; 3 Department of Mechanical Engineering, Madhav Institute of Technology and Science, Gwalior 474005, India; amit.aherwar05@mitsgwalior.in; 4 Mechanical Engineering Department, Graphic Era (Deemed to be University), Dehradun 248002, Uttarakhand, India; lalit_9000@yahoo.com; 5 Department of Materials Science and Engineering, Széchenyi István University, 9026 Győr, Hungary; dogossy@sze.hu

**Keywords:** PLA biocomposite, wood waste, rice husk, physicomechanical, wear, CRITIC–MABAC

## Abstract

Based on the criteria importance through inter-criteria correlation (CRITIC) and the multi-attributive border approximation area comparison (MABAC), a decision-making algorithm was developed to select the optimal biocomposite material according to several conflicting attributes. Poly(lactic acid) (PLA)-based binary biocomposites containing wood waste and ternary biocomposites containing wood waste/rice husk with an overall additive content of 0, 2.5, 5, 7.5 and 10 wt.% were manufactured and evaluated for physicomechanical and wear properties. For the algorithm, the following performance attributes were considered through testing: the evaluated physical (density, water absorption), mechanical (tensile, flexural, compressive and impact) and sliding wear properties. The water absorption and strength properties were found to be the highest for unfilled PLA, while modulus performance remained the highest for 10 wt.% rice husk/wood-waste-added PLA biocomposites. The density of PLA biocomposites increased as rice husk increased, while it decreased as wood waste increased. The lowest and highest density values were recorded for 10 wt.% wood waste and rice husk/wood-waste-containing PLA biocomposites, respectively. The lowest wear was exhibited by the 5 wt.% rice husk/wood-waste-loaded PLA biocomposite. The experimental results were composition dependent and devoid of any discernible trend. Consequently, prioritizing the performance of PLA biocomposites to choose the best one among a collection of alternatives became challenging. Therefore, a decision-making algorithm, called CRITIC–MABAC, was used to select the optimal composition. The importance of attributes was determined by assigning weight using the CRITIC method, while the MABAC method was employed to assess the complete ranking of the biocomposites. The results achieved from the hybrid CRITIC–MABAC approach demonstrated that the 7.5 wt.% wood-waste-added PLA biocomposite exhibited the optimal physicomechanical and wear properties.

## 1. Introduction

With rapid urbanization, industrialization and population growth, the production of wastes and by-products is increasing continuously. These wastes become the cause of many health and ecological issues, in case they are not treated properly; therefore, their disposal emerges as a significant problem [1]. Most countries have issues with discarded waste (municipal, agricultural and forestry), and it is critical to make good use of the available land for infrastructure, agriculture, industry and other purposes [1,2]. Open-space dumping and landfilling are the primary sources of pollution globally. Almost 37% of the municipal waste is disposed of in some type of landfill, while 31% is disposed of via open dumping [3]. The carbon in these organic wastes could be released as harmful carbon dioxide, leachates or volatile organic carbons. In 2016, the solid waste treatment and disposal produced 1.6 billion tons of carbon-dioxide-equivalent greenhouse gas emissions, and this is projected to increase by 63% to reach 2.6 billion tons by 2050 [3]. Instead of landfilling and open dumping, nowadays, nations promote the recycling/reusing of wastes to curb environmental pollution [4,5]. As a result, scientists are continually making extreme efforts to use municipal, agricultural and forest industry waste in useful applications, which facilitates not only reducing environmental pollution but also lowering manufacturing costs [6,7,8,9]. One of the impending applications is polymer biocomposites, where incorporating agricultural or forest industry waste has proven to be quite beneficial from the techno-commercial and ecological point of view [10,11,12,13]. As a result, in recent years, the use of agricultural and forest industry waste in the manufacturing of biocomposites has gained popularity and has become a regular practice [14,15,16]. In this regard, the literature is rich in research where agricultural and wood wastes were used to manufacture biocomposites using different biopolymeric matrices, such as polyhydroxyalkanoates, poly(lactic acid) (PLA), polybutylene succinate, poly(butylene adipate terephthalate), etc. [17,18,19,20]. PLA is an aliphatic polyester that can be manufactured from renewable resources, and it is one of the most important members of the biopolymer family [21]. Furthermore, PLA is completely biodegradable, and the by-products being generated during its decomposition are non-toxic. PLA is regarded as a valuable substitute for synthetic polymers in a variety of applications, including packaging, healthcare and textile, because of its strong mechanical qualities, high stiffness and biodegradability [21,22]. The high cost and low thermal stability are a few shortcomings that limit the use of PLA as a commodity polymer for large-scale applications [23,24]. A viable solution for these downsides includes blending with different biopolymers or the addition of agricultural and forest industry waste as cheap fillers in PLA-based biocomposites [25].

Baynast et al. [26] studied the influence of two different by-products of the forest (Kraft lignin) and agriculture (corn cob) industries on the thermal and mechanical performance of PLA-based biocomposites. It was found that both added waste fillers acted as nucleating agents by facilitating the cold crystallization of PLA, whereas thermal stability was found to decrease with filler addition. The highest hardness was recorded for 20 wt.% Kraft lignin added PLA biocomposite, which was almost relatively 47% higher compared to unfilled PLA. The biocomposite containing a mixture of Kraft lignin (2.5 wt.%) and corn cob (9 wt.%) exhibited the highest flexural strength of 84 MPa, while flexural modulus remained highest (3.7 GPa) for the biocomposite, with 2.5 wt.% Kraft lignin and 9 wt.% corn cob. The applicability of cocoa bean shell in PLA biocomposites was investigated by Garcia-Brand et al. [27]. The authors concluded that crystallinity increased with the addition of 2.5 wt.% cocoa bean shell, while a small decrement in glass transition temperature was noticed. Moreover, the tensile strength and elongation of the manufactured composite decreased, while Young’s modulus and toughness increased with the incorporation of 2.5 wt.% cocoa bean shell. Wu and Tsou [28] studied the influence of rice husk on the mechanical and water absorption performance of PLA composites. The authors concluded that the tensile strength decreased while the water absorption rose with increased rice husk concentration. Nizamuddin et al. [29] extensively investigated the physicomechanical and thermal aspects of rice-husk-char (0–20 wt.%)-loaded PLA composites. The tensile strength of the composites decreased from 43.69 MPa to 36.82 MPa, while the tensile modulus increased from 2.63 GPa to 4.24 GPa as the concentration of rice husk char increased from 0 wt.% to 20 wt.%. While studying the applicability of wood waste (0 to 40 wt.%) in PLA composites, Boubekeur et al. [30] concluded that the Young’s modulus remained the largest for 40 wt.% added composites, while a 10 wt.% wood-waste-added composite resulted in the second best impact strength. The impact of hybrid cork wood (0 to 30 wt.%) waste in PLA composites was investigated by Andrzejewski et al. [31]. The authors concluded that the tensile strength and modulus values decreased with hybrid filler loadings. The impact strength of unfilled PLA was found to be the highest (4.5 kJ/m^2^), and it decreased to ~2 kJ/m^2^ without any pronounced trend as a result of cork-wood waste filling. The influence of chestnut shell (2.5 to 30 wt.%) waste on the mechanical properties of PLA composites was investigated by Barczewski et al. [32]. The highest elastic modulus was recorded for 30 wt.% chestnut-shell-waste-added PLA composites, while the largest values of tensile and impact strength were exhibited by the PLA composite with 2.5 wt.% waste content.

In general, many properties of PLA biocomposites increase with the inclusion of waste reinforcement. However, this enhancement in properties is affected by the type and amount of the incorporated fibers/particles. Moreover, the fabricated composite has its own performance for each individual characteristic; thus, a decision on the optimal composition with the highest degree of fulfillment for all material characteristics is essential. This can be accomplished using the multi-attribute decision-making (MADM) analysis [33,34]. MADM approaches are the systematic ways for determining the importance of attributes (material properties) by examining a specific application and selecting the best candidate material, while eliminating the inappropriate alternatives [35,36]. Various MADM approaches, such as AHP, GRA, CRITIC, TOPSIS, VIKOR, SAW, MEW, COPRAS, PSI and ELECTRE, etc., have been created to assist in rating material alternatives and picking the best one [37,38,39,40,41,42,43,44]. Among them, MABAC and CRITIC are two popular ones. The MABAC method is an outranking technique based on the distance of alternatives from a border approximate area matrix. Meanwhile, the MABAC technique is a very efficient ranking method due to its basic mathematical computations and ease of usage compared to other MADM techniques [42]. Unlike MABAC, CRITIC addresses how to determine the relative importance of a set of attributes in any MADM technique by applying the inter-criteria correlation [43]. Either of the two approaches is suitable for particular MADM problems and has successfully been applied in various engineering and management domains [41,42,43,44].

In this research, an attempt was made to obtain an optimized composition of PLA biocomposite materials filled with wood waste and hybrid wood waste/rice husk. In order to do this, PLA-based biocomposites reinforced with wood waste and hybrid wood waste/rice husk (0, 2.5, 5, 7.5 and 10 wt.%) were developed and characterized for various physical (density and water absorption), mechanical (tensile strength and modulus, compressive strength and modulus, flexural strength and modulus, impact strength) and wear properties. The highest filler amount was restricted to 10 wt.%, as the literature reported that not only does the process ability become difficult during extrusion and injection molding, but properties such as toughness and strength also decrease with increased filler loading [45]. The experimental results were found to be strongly composition dependent and without any apparent trends. As a result, it becomes difficult to prioritize the biocomposites or select one as the best by simultaneously considering all the evaluated properties. Therefore, by fixing the evaluated properties as selection attributes, the optimization was conducted in two steps of weight determination by the CRITIC method and the MABAC method to rank the biocomposite alternatives.

## 2. Materials and Methods

### 2.1. Materials and Biocomposite Fabrication

**Figure 1:**
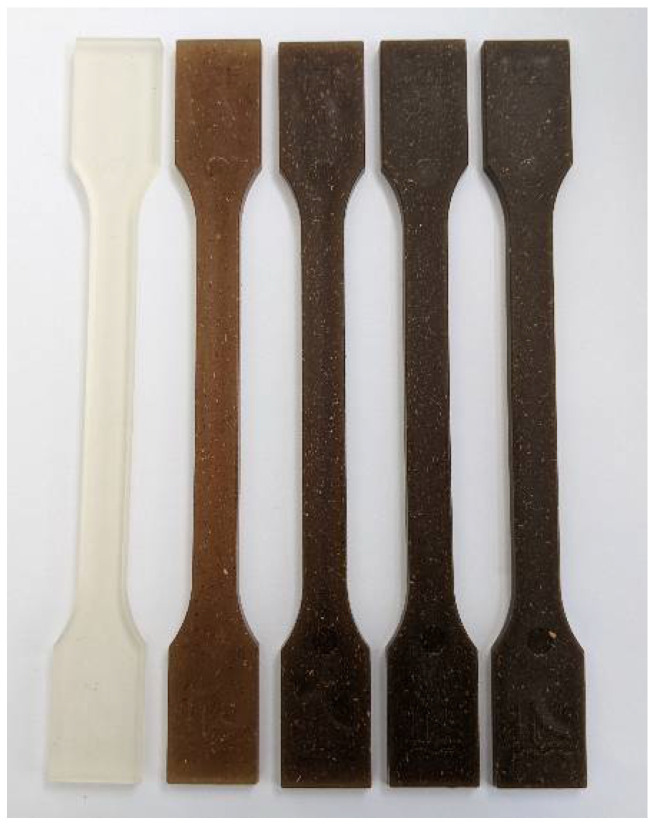
The fabricated specimens.

Poly(lactic acid) (PLA) was supplied by Nature Works, USA (Ingeo 2003D grade) in a pellet form. The density and melting temperature of this PLA grade were 1.24 g/cm^3^ and 170 °C, respectively. Wood flour waste and rice husk were obtained from the Krishna Timber Store and a local farmer, Himachal Pradesh (India), respectively. The obtained fillers were sieved to achieve a particle size of 60 mesh. Thereafter, the fillers’ surface was modified using alkali treatment (2 wt.% sodium hydroxide solution) at room temperature for 12 hours. After washing them with distilled water four times, the treated fillers were oven dried for 4 hours at 80 °C. The PLA granules, wood waste and rice husk were dried in a DEGA-2500 (DE.GA S.p.A., Corte Franca, Italy) type dryer at 80 °C for 6 hours prior to melt processing in order to remove their inherent moisture content and thereby to prevent the hydrothermal degradation of the polymer during the melt processing. The PLA biocomposites were fabricated by melt compounding and injection molding using the compositional variations presented in Table 1. Melt mixing was performed using a co-rotating twin-screw extruder (Labtech LTE 20-44 type, Samut Prakarn, Thailand) with a rotational speed of 30 rev/min. The screw diameter and L/D ratio were 20 mm and 44, respectively. From the feeder to the die, the extruder barrel temperature was varied from 155 °C to 185 °C. Before further processing, the extruded materials were pelletized and dried at 80 °C for 6 hours. Subsequently, the test samples (Figure 1) were molded into dogbone-shaped specimens (EN ISO 527-2 type A), using an Arburg Allrounder Advance injection molding machine (420C Golden Edition; Arburg, Germany) with a screw of 35 mm diameter. The nozzle temperature of the injection unit was 195 °C, while the injection rate was 40 cm^3^/s. The holding pressure profile was set to 750-650-250 bar (for a total of 15 s). The mold temperature was set to 35 °C, with a residual cooling time of 30 s.

### 2.2. Measurements

The wood waste and wood/rice husk-waste-filled PLA biocomposites were measured for various mechanical (tensile, compressive, flexural and impact), physical (density, water absorption) and sliding wear properties. These were considered as the performance attributes in the ranking process. The density of the composites was computed using the Archimedes principle by weighing the samples in the air and then in water. The water absorption known weight (let W_1_) of composite samples was engrossed in deionized water for 5 days. Subsequently, the samples were taken out and weighed (let W_2_) after having been cleaned with a tissue paper. The following formula was used for the estimation of water absorption (%) [30]:

(1) $$\mathrm{Water}\;\mathrm{absorption}\;(\%)=\frac{W_{2}-W_{1}}{W_{1}}\times 100$$

The three-point bending, tensile and compression tests of the manufactured PLA-based samples were performed using the Instron 5582 testing machine (Instron Ltd., Norwood, MA, USA). The three-point bending and tensile tests were performed using 5 mm/min of cross-head speed (1 mm/min speed for modulus). As-fabricated dogbone biocomposite samples (170 mm × 10 mm × 4 mm) were used for tensile testing. In contrast, rectangular samples (80 mm × 10 mm × 4 mm) were used for three-point bending. The clamping distance for tensile testing was 100 mm, while the span length for three-point bending was 64 mm. The compression test was carried out with rectangular samples of 10 mm × 4 mm cross-section. For the compression modulus, samples of 50 mm height were used with 1 mm/min of compression speed. As for compressive strength, the sample height was 10 mm, and the speed was 5 mm/min. The Charpy impact strength of the unnotched biocomposite samples (80 mm × 10 mm × 4 mm) was measured using the Ceast 6545 (Ceast, Pianezza, Italy) impact testing machine equipped with a hammer of 2 J impact energy and a span length of 62 mm. The dry sliding wear test was executed using a pin-on-disc machine (TR-411, DUCOM, India) to determine the wear loss of fabricated PLA biocomposites at ambient room temperature, as per ASTM G-99 standard. During the wear test, the biocomposite sample was held stationary, while the hardened alloy steel disc was rotated, and the load was applied through a lever mechanism. The wear tests were performed for fixed conditions of load = 50 N, track diameter = 50 mm, sliding distance = 2.5 km and sliding velocity = 3 m/s. The wear in terms of weight loss (in gram) was computed using the following formula:

(2) $$\mathrm{Wear}\;(g)=\frac{{Χ}_{2}-{Χ}_{1}}{{Χ}_{1}}\times 100$$

where ${Χ}_{1}$ = sample weight before wear test, ${Χ}_{2}$ = sample weight after wear test.

The surfaces exposed to the sliding wear tests were studied with scanning electron microscopy (SEM). For this purpose, an S-3400N (Hitachi, Tokyo, Japan) scanning electron microscope with an acceleration voltage of 10 kV was applied. The surfaces were sputter coated prior to SEM inspection using an SC7620 sputter coater (Quorum Technologies Ltd., Laughton, UK).

To check the consistency of the obtained results, the wear experiments were repeated three times, while for physical and mechanical characterization, experiments were repeated five times at ambient temperature. The performance implications and test conditions of the selected attributes are provided in Table 2.

## 3. Overview of the Integrated CRITIC–MABAC Approach

**Figure 2:**
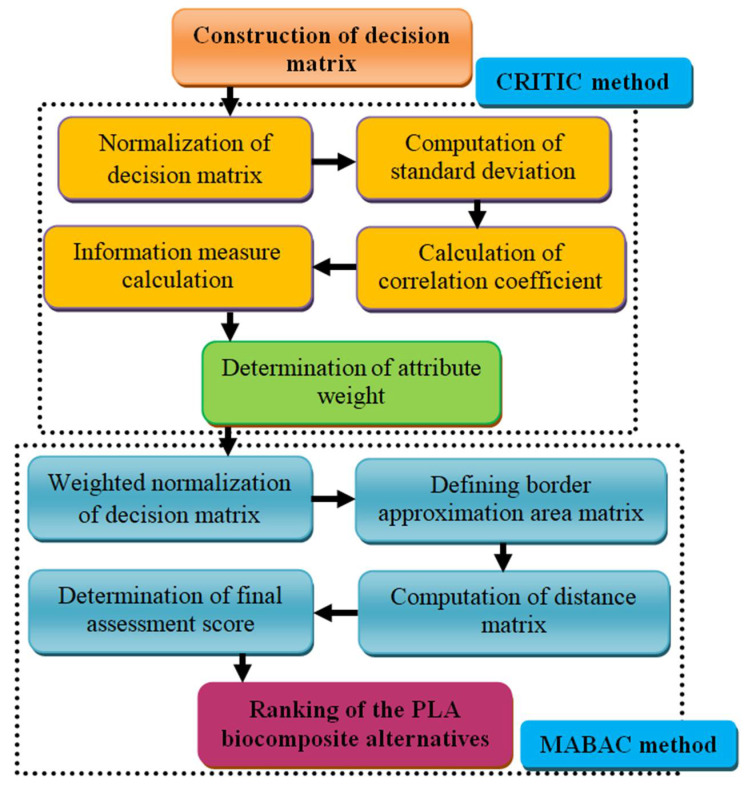
Proposed CRITIC–MABAC algorithm.

An integrated CRITIC–MABAC technique was proposed to perform a performance evaluation and to rank the best alternatives of wood waste and wood/rice husk-waste-filled PLA composites. Figure 2 depicts a schematic diagram of the approach used in the suggested methodology. The process is concerned with determining the attribute weight using the CRITIC and the best alternatives using the MABAC method. Diakoulaki et al. [46] provide the CRITIC method as an objective tool for calculating the attribute weight in a given MADM strategy. Standard deviation of the attributes and correlation analysis are used in the CRITIC technique. In addition to being popular and simple, researchers have also proposed modifications to increase its reliability and accuracy. Wu et al. [47] applied Z-transformation-based normalization to modify the conventional CRITIC approach for urban railway transit operations. Krishnan et al. [48] used a distance correlation concept to introduce the D-CRITIC weighting approach to tackle more significant MADM problems. Haktanır and Kahraman [49] proposed picture-fuzzy-sets-based CRITIC methodology for wearable health technology selection problems. 

Pamučar and Ćirović [50] proposed the MABAC approach, a newly designed MADM method. Building a border approximation area matrix and evaluating its distance from each alternative is one of the MABAC method’s main principles [40,41,44]. The various advantages of the MABAC approach are its easy computational procedure, consistency in the computed results and use of both the profit and loss values of attributes in the computational process. In addition to being a simple, popular and organized computational approach, researchers have proposed modifications to increase the reliability and accuracy of MABAC in real-world decision-making problems. Yu et al. [51] proposed interval type-2 fuzzy-numbers-modified MABAC approach for making hotel selection. Sun et al. [52] provided hesitant-fuzzy-linguistic-term-sets-modified MABAC model for patients’ prioritization. Xu et al. [53] used linguistic-hesitant-fuzzy-sets, real-numbers, interval-numbers and trapezoidal-fuzzy-numbers-based MABAC approach to solve the green supplier evaluation and selection problem. Wang et al. [54] combined the conventional MABAC approach with a q-rung orthopair fuzzy set to solve MADM problems. Huang et al. [55] proposed Z-cloud rough-numbers-integrated MABAC approach for design selection. Hesitant-fuzzy-linguistic and D-numbers-based weighting approaches were also used to derive the MABAC model by Büyüközkan et al. [56] and Pamučar et al. [57], respectively. More recently, Simic and co-workers [58] used type-2 neutrosophic-numbers-derived CRITIC- and MABAC-based integrated approach for selecting the optimal public transportation pricing system. 

The hybrid CRITIC–MABAC technique has the following process steps:

*Step 1:* A decision matrix is formulated, as presented in Equation (3).

(3) $$A_{p\times q}=\left|\begin{array}{cccccc} A_{11} & A_{12} & \ldots & A_{1j} & \ldots & A_{1q}\\ A_{21} & A_{22} & \ldots & A_{2j} & \ldots & A_{2q}\\ \vdots & \vdots & \ldots & \vdots & \ldots & \vdots \\ A_{i1} & A_{i2} & \ldots & A_{ij} & \ldots & A_{iq}\\ \vdots & \vdots & \ldots & \vdots & \ldots & \vdots \\ A_{p1} & A_{p2} & \ldots & A_{pj} & \ldots & A_{pq} \end{array}\right|\begin{array}{l} i=1,\;\;2,\cdots ,\;\;p\\ j=1,\;\;2,\cdots ,\;\;q \end{array}$$

where *p* is the alternatives number; *q* is the attributes number; *A_ij_* is the presentation of *i*th alternative with respect to the *j*th attribute.

*Step 2:* According to the orientation of attributes, normalization is performed using the following formulae:

(4) $${\bar{A}}_{ij}=\frac{A_{ij}-\min(A_{j})}{\max(A_{j})-\min(A_{j})},\;\mathrm{if}\;j\in \;\mathrm{benefit}\;(\mathrm{higher}-\mathrm{the}-\mathrm{better})\;\mathrm{attribute}\;{\bar{A}}_{ij}=\frac{\max(A_{j})-A_{ij}}{\max(A_{j})-\min(A_{j})},\;\mathrm{if}\;j\in \;\cos t\;(\mathrm{lower}-\mathrm{the}-\mathrm{better})\;\mathrm{attribute}$$

*Step 3:* The correlation coefficient ($\sigma_{jk}$) among the various attributes determined as

(5) $$\sigma_{jk}=\frac{\sum_{i=1}^{p}\left({\bar{A}}_{ij}-\Delta_{j}\right)\;\left({\bar{A}}_{ik}-\Delta_{k}\right)}{\sqrt{\sum_{i=1}^{p}{\left({\bar{A}}_{ij}-\Delta_{j}\right)}^{2}\sum_{i=1}^{p}{\left({\bar{A}}_{ik}-\Delta_{k}\right)}^{2}}}$$

where $\Delta_{j}$ and $\Delta_{k}$ are the mean of *j*th and *k*th attributes. The $\Delta_{j}$ is computed using the following equation:

(6) $$\Delta_{j}=\frac{1}{p}\sum_{i=1}^{p}{\bar{A}}_{ij};\;j=1,\cdots ,q$$

Similarly, $\Delta_{k}$ is computed by replacing *j* with *k* in Equation (6).

*Step 4:* The information measure ($\chi_{j}$) value of each attribute is determined using the following equation:

(7) $$\chi_{j}=\partial_{j}\sum_{k=1}^{q}\left(1-\sigma_{jk}\right)\;;\;j=1,\cdots ,q$$

Here, $\partial_{j}$ represents the standard deviation of the *j*th attribute. 

*Step 5:* Determine the attribute weight ($\varpi_{j}$) using the following formula:

(8) $$\varpi_{j}=\frac{\chi_{j}}{\sum_{j=1}^{q}\chi_{j}};\;j=1,\cdots ,q$$

where $\varpi_{j}\;\left(j=1,2,\ldots ,q\right)$ is the attribute weight with $\varpi_{j}\in \left[0,\;1\right]$ and $\sum_{j=1}^{q}\varpi_{j}=1.$*Step 6:* The weighted normalized decision matrix ($w={\left[w_{ij}\right]}_{p\times q}$) is formulated using the following formula:

(9) $$w_{ij}=\varpi_{j}({\bar{A}}_{ij}+1),\;i=1,\cdots ,p;\;j=1,\cdots ,q$$

*Step 7:* Border approximation area (${Β}_{j}$) matrix is determined using the following formula:

(10) $${Β}_{j}={\left(\prod_{i=1}^{p}w_{ij}\right)}^{1/p},\;j=1,\cdots ,q$$

*Step 8:* The distance matrix $\left(D={\left[D_{ij}\right]}_{p\times q}\right)$ is defined using following the formula:

(11) $$D_{ij}=w_{ij}-{Β}_{j},\;i=1,\cdots ,p;\;j=1,\cdots ,q$$

where $D_{ij}$ represents the distance of *i*^th^ alternative (*A_i_*) from the border approximation area (${Β}_{j}$) matrix under *j*^th^ attribute.

*Step 9:* The assessment score ($\Phi_{i}$) for each alternative is determined using the following formula:

(12) $$\Phi_{i}=\sum_{j=1}^{q}D_{ij},\;\;i=1,\cdots ,p$$

The alternatives are ranked according to the obtained values of the assessment score ($\Phi_{i}$). The best alternative is one with the highest $\Phi_{i}$ value.

## 4. Results and Discussion

### 4.1. Influence of Waste Loading on the Performance of Various Attributes

The results of wood waste and wood/rice husk waste loading on the performance of each attribute are presented in Table 3. Table 3 consists of nine biocomposite alternatives (p-1 to p-9, as described in Table 1) and ten properties (q-1 to q-10, as described in Section 2.2) fixed as selection attributes. The selection attributes include tensile strength (q-1), compressive strength (q-2), impact strength (q-3), flexural strength (q-4), tensile modulus (q-5), compressive modulus (q-6), flexural modulus (q-7), density (q-8), water absorption (q-9) and wear (q-10). The results demonstrate that the wood waste and rice husk loading significantly affect the evaluated performance attributes of the PLA-based biocomposites. The tensile strength of the biocomposite alternatives (attribute q-1) decreased both with an increased wood waste and wood/rice husk waste loading. The tensile strength peaked at 57.96 MPa for the p-1 alternative and decreased relatively by 9% to 53 MPa for the p-6 (2.5 wt.% wood waste) alternative, while for other biocomposites, it fluctuated in the range of 51.96 ± 0.90 MPa, and it remained the lowest (50.06 MPa) for the alternative p-4, with 7.5 wt.% wood waste/rice husk. This drop in tensile strength with increased filler loadings might be attributed to the irregularly shaped wood waste/rice husk particles, acting as stress concentration sites within the PLA matrix. Moreover, the chances of wood waste/rice husk particles agglomerations with an increased filler loading resulted in their decreased adhesion to PLA resin. Similar results for strength decrement with lignocellulosic materials are reported for various polymeric composites. Wu and Tsou [28] reported that the tensile strength of neat PLA decreased from 44.8 MPa to ~38 MPa with the addition of 10 wt.% rice husk. Quiles-Carrillo et al. [59] reported that the tensile strength of PLA decreased from 63.3 MPa to 39.7 MPa with almond shell flour. Sánchez-Safont et al. [60] reported that the tensile strength of poly(3-hydroxybutyrate) decreased by 5 to 10 MPa with the inclusion of rice husk, almond shell and seagrass wastes. Another study by Pudełko et al. [61] reported that the tensile strength of PLA decreased by almost 36%, from 67 MPa to 43 MPa, with the addition of 10 wt.% wood-waste-derived biochar. 

Consequently, the wood waste/rice husk not only reduced the tensile strength but also resulted in a detrimental impact on the compressive strength (attribute q-2) and the impact strength (attribute q-3) of the PLA biocomposites. Similar results for reduced strength with increased natural fiber are well described in the literature [30,31]. The highest compressive strength of 105.67 MPa and the impact strength of 15.25 kJ/m^2^ were exhibited by unfilled PLA, i.e., p-1 alternative. The compressive and the impact strengths decreased gradually with the waste loading, and they fluctuated in the range of 100.78 ± 1.34 MPa and 10.6 ± 1.8 kJ/m^2^, respectively. The compressive strength decreased relatively by ~6% to the lowest value of 99.44 MPa for the PLA biocomposite containing 2.5 wt.% wood waste, i.e., p-6 alternative, while the impact strength decreased relatively by ~42% to the lowest value of 8.80 kJ/m^2^ for 7.5 wt.% wood waste/rice husk added, i.e., p-4 alternative. Corresponding results for decreased impact strength of PLA biocomposites with increased wood-waste-derived biochar content were reported by Pudełko et al. [61]. It has been reported that the impact strength of unfilled PLA was decreased relatively by ~31% from 13 kJ/m^2^ to 9 kJ/m^2^ for 10 wt.% biochar-added biocomposite and decreased relatively by ~73% to 3.5 kJ/m^2^ with further addition (20 wt.%) of wood-waste-derived fiber biochar.

The largest (100.43 MPa) and the smallest (97.4 MPa) flexural strength (attribute q-4) were exhibited by p-7 and p-5 alternatives, respectively, and they fluctuated in a small range of 98.9 ± 1.5 MPa. Overall, the flexural strength of wood waste/rice husk filled PLA biocomposites was comparable to that of the unfilled PLA, suggesting that rice husk and/or wood waste have a great potential as fillers/reinforcements in polymer composites. These results are in strong agreement with the literature, as the addition of natural plant fillers was reported to reduce the flexural strength of the biocomposites [2]. Contrary to the strength properties, the tensile modulus (attribute q-5), the compressive modulus (attribute q-6) and the flexural modulus (attribute q-7) of the biocomposites grew with an increased wood waste/rice husk loading. The lowest values of tensile modulus (2.56 GPa), compressive modulus (2.71 GPa) and flexural modulus (3.43 GPa) were exhibited by the unfilled PLA. On the other hand, the largest tensile modulus (3.02 GPa), compressive modulus (3.58 GPa) and flexural modulus (4.03 GPa) values were recorded for 10 wt.% wood waste/rice husk added PLA biocomposites, i.e., p-5 alternative. The tensile, compressive and flexural modulus of the unfilled PLA increased relatively by ~18%, ~32% and 17%, respectively, when adding 10 wt.% wood waste/rice husk to it. This improvement can be attributed to the high stiffness of lignocellulosic fillers, and the results were in line with the published literature [62,63]. Quiles-Carrillo et al. [59] reported that the unfilled PLA’s tensile and flexural modulus increased by 30% and 26%, respectively, with almond shell flour. Garcia-Brand et al. [27] reported that the tensile modulus of neat PLA increased from 3.33 GPa to 3.45 GPa with the inclusion of cocoa bean shell waste. Das et al. [64] claimed that the high surface area of the lignocellulosic fillers promoted stress transfer between the polymer matrix and the filler particles, resulting in a reduction in the deformation of the polymer and an improvement in the modulus.

The results show that the density (attribute q-8) rose gradually with increasing hybrid wood waste/rice husk, while it constantly decreased with increasing wood waste loading. This trend in increasing density with wood waste/rice husk was anticipated due to the use of high-density rice husk content to replace the low-density PLA. Meanwhile, the decrease in the density of wood-waste-filled PLA biocomposites was attributed to the lower density of wood waste compared to PLA [65]. The highest density of 1.277 g/cm^3^ was recorded for the p-5 alternative, with 10 wt.% wood waste/rice husk, while density remained the lowest (1.183 g/cm^3^) for the p-9 alternative, with 10 wt.% wood waste content. The water absorption (attribute q-9) of the PLA biocomposites grew with the increased rice husk/wood waste loading. The lowest water absorption (0.36%) was recorded for unfilled PLA, i.e., p-1 alternative, while it remained the largest (1.92%) for 10 wt.% wood-waste-loaded biocomposite, i.e., for p-9 alternative. The inclusion of lignocellulosic fillers was extensively reported to enhance the water absorption of polymeric composites. The increment in water absorption for PLA-based composites is expected with the addition of lignocellulosic fillers and is in line with the published literature [30,61]. This is because lignocellulosic fillers are mainly composed of cellulose, lignin, hemicellulose and other polysaccharides with strong water-binding and swelling ability. Boubekeur et al. [30] reported that the water absorption of unfilled PLA increased from 1.05% to 1.78% with the inclusion of 10 wt.% waste wood flour, and it increased further to 12.38% with 40 wt.% wood-flour-loaded composites. Pudełko et al. [61] reported that the water absorption of unfilled PLA was 0.5% and increased to 1.51% for 10 wt.% and to 1.99% for 20 wt.% wood-waste-derived biochar-added biocomposites. 

The wear (attribute q-10) of the PLA biocomposites was found to decrease with low wood waste/rice husk (≤5 wt.%) and wood waste (≤2.5 wt.%) loadings, and it was found to increase with further rice husk/wood waste loading. The wear was 0.1652 g for unfilled PLA. It decreased relatively by ~39% to the lowest value of wear, 0.1014 g, for the PLA-based biocomposite containing 5 wt.% wood waste/rice husk, i.e., p-3 alternative. In comparison, the most extensive wear of 0.2618 g was exhibited by the p-9 biocomposite alternative, with 10 wt.% wood waste content. The lower rice husk and/or wood waste loading results in their uniform distribution and helps the matrix withstand more distortion during sliding, resulting in a reduced wear compared to unfilled PLA. With an increased filler loading (≥7.5 wt.%), the possible agglomeration and uneven distribution of rice husk and/or wood waste in the matrix resulted in their easy removal from the PLA resin, and it increased the wear of the biocomposite, as found experimentally [66]. Mysiukiewicz et al. [67] concluded that the wear performance of linseed-cake-filled and natural-oil-lubricated PLA composites remained comparable to neat PLA. Snowdon et al. [68] reported that the wear resistance of neat PLA increased with the addition of biocarbon derived from pyrolyzed *Miscanthus*. Bajpai et al. [69] extensively studied the effect of lignocellulosic fibers, namely *Grewia optiva*, nettle and sisal, on the wear performance of PLA-based composites under various load-speed conditions. The authors claimed that the wear rate of unfilled PLA decreased relatively by ~70% with the addition of lignocellulosic fibers.

**Figure 3:**
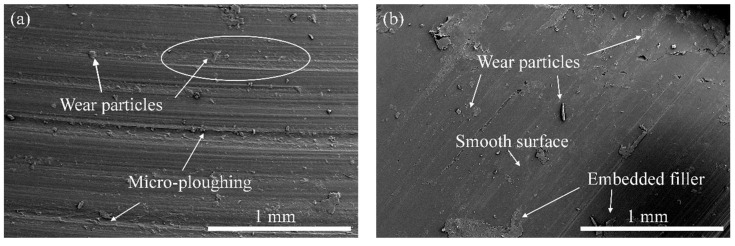

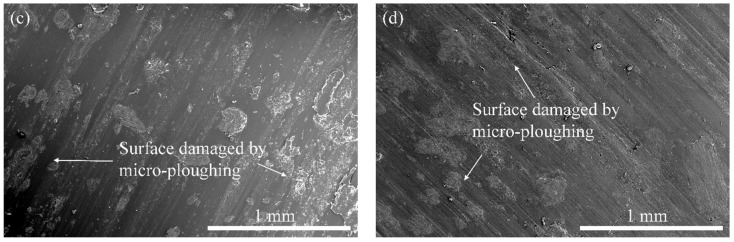
Worn micrographs of biocomposite alternatives; (**a**) p-1, (**b**) p-2, (**c**) p-8 and (**d**) p-9.

The observed trends in wear were evident by studying the worn surfaces of the composites, as presented in Figure 3. The wear of the unfilled PLA was mainly caused by extensive micro-ploughing due to the softening process of the matrix during sliding. The inclusion of the lower rice husk and/or wood waste (≥5 wt.%) particles helps the PLA matrix withstand the heat generated during sliding, and it keeps the matrix from being removed. The worn composite surface appeared smoother with fewer signs of micro-ploughing, suggesting a reduced wear of the composites. For composites with high filler loading (>5 wt.%), the worn surface looks heavily deformed with scattered wear particles. Apart from their uneven distribution, the number of rice husk and/or wood waste particles on the surface also increased, and they might be easily detached from the composites during sliding, resulting in more wear.

**Figure 4:**
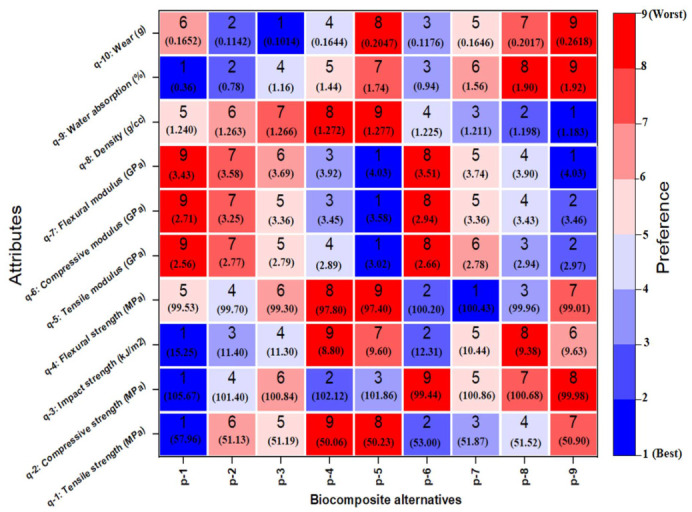
Preference order of biocomposite alternatives for selected attributes.

The results of PLA biocomposite alternatives can be arranged in performance orders concerning each attribute, as illustrated in Figure 4.

The results clearly reveal that no single PLA biocomposite alternative has better performance concerning all attributes at a time. However, it should be noted that the tensile strength (attribute q-1), compressive strength (attribute q-2), impact strength (attribute q-3) and water absorption (attribute q-9) of p-1 are superior among all biocomposite alternatives, while having the poorest tensile modulus (attribute q-5), compressive modulus (attribute q-6) and flexural modulus (attribute q-7). The alternative p-7 exhibits flexural strength (attribute q-4), while alternative p-3 records the best wear (attribute q-10) performance. The tensile modulus (attribute q-5), compressive modulus (attribute q-6) and flexural modulus (attribute q-7) of p-5 are the best among all biocomposite alternatives; however, they exhibit the lowest impact strength (attribute q-3) and the poorest density (attribute q-8). The results uncover no particular biocomposite alternative that shows the best solution considering all attributes simultaneously. Hence, it is difficult to suggest an option for which the biocomposite would deliver the highest performance. Therefore, the hybrid CRITIC–MABAC approach is utilized to rank these biocomposites.

### 4.2. CRITIC Analysis for Weight Calculation

The objective weights of the attributes used in PLA biocomposite ranking were computed using the CRITIC method. Table 3 shows the decision matrix of nine PLA biocomposite alternatives that were compared based on ten attributes, namely tensile strength (q-1), compressive strength (q-2), impact strength (q-3), flexural strength (q-4), tensile modulus (q-5), compressive modulus (q-6), flexural modulus (q-7), density (q-8), water absorption (q-9) and wear (q-10). Table 4 shows the normalized decision matrix derived from Equation (4). For the implication of Equation (4), the benefit (higher-the-better) and the cost (lower-the-better) values of each attribute were carefully identified. For a benefit attribute, the highest value is taken as the best one, while for a cost attribute, the smallest value is considered as the best one and vice versa for the worst values. For example, for a benefit attribute (tensile strength; q-1), the best and worst values are 57.96 MPa for p-1 and 50.06 MPa for p-4, respectively. Meanwhile, for a cost attribute (wear; q-10), 0.1014 g and 0.2618 g are considered the best and the worst values, respectively.

The information measure values ($\chi_{j}$) of each attribute were determined using Equation (7), and they are listed in Table 5. These $\chi_{j}$ values suggest that the data pattern of flexural modulus (q-7) has the highest information value (4.1872), followed by tensile modulus (q-5; 3.6803), and it remains the lowest for tensile strength (q-1; 2.5713). Furthermore, the weight ($\varpi_{j}$) of various attributes was also determined using Equation (8) and presented in Table 5. Based on Table 5, the attribute q-7 (flexural modulus) is identified as the most important attribute with a weight score of 0.1306, while attribute q-1 (tensile strength) was given the lowest priority with a weight score of 0.0802.

### 4.3. MABAC Analysis for Alternatives Ranking

After calculating the attributes’ weight using the CRITIC approach, the MABAC method was implemented for the ranking of PLA biocomposite alternatives. The weighted normalized decision matrix was structured using Equation (9) and is provided in Table 6. Thereafter, the border approximation area (${Β}_{j}$) matrix was structured using Equation (10), as listed in Table 7. For q-1 attribute, the ${Β}_{j}$ matrix value was determined as

$${Β}_{1}={\left(0.1604\times 0.0911\times 0.0917\times 0.0802\times 0.0819\times 0.011\times 0.0986\times 0.095\times 0.0887\right)}^{1/9}=0.0976$$

Similarly, ${Β}_{j}$ can be determined for other attributes, listed in Table 7.

Following that, the distance ($D_{ij}$) of each alternative of the weighted matrix from the structured ${Β}_{j}$ matrix was determined using Equation (11) and presented in Table 8. For example, the distance of alternatives from the attribute q-1 of ${Β}_{j}$ matrix was computed as follows:

$$\begin{array}{l} D_{11}=0.1604-0.0976=0.0628\\ D_{21}=0.0911-0.0976=-0.0065\\ D_{31}=0.0917-0.0976=-0.0059\\ \vdots \\ D_{81}=0.0950-0.0976=-0.0026\\ D_{91}=0.0887-0.0976=-0.0089 \end{array}$$

Finally, the assessment score ($\Phi_{i}$) for each biocomposite alternative was computed using Equation (12). For p-1 alternative, the $\Phi_{i}$ value was computed as the following:

$$\Phi_{1}=0.0628+0.0571+0.0577+0.0121-0.0611-0.0709-0.0661+0.0013+0.0623+0.0036=0.0588$$

**Figure 5:**
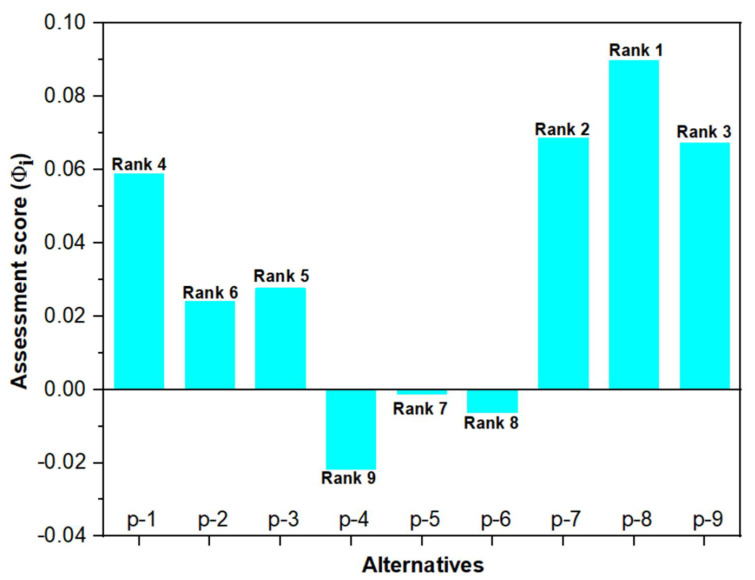
Ranking of PLA biocomposite alternatives.

The computed $\Phi_{i}$ values and the corresponding ranking of the PLA biocomposite alternatives are presented in Figure 5. From Figure 5, it can be observed that the $\Phi_{i}$ of the alternative p-8 is the highest (0.0898), signifying the best one (Rank 1) among all the available PLA biocomposite alternatives. However, p-8 alternative is followed by alternatives p-7 (0.0687) and p-9 (0.0674), whereas the $\Phi_{i}$ of the biocomposite alternative p-4 was the smallest (−0.0218). From the hybrid CRITIC–MABAC analysis, it was observed that the biocomposite p-8 with 7.5 wt.% wood waste exhibited the optimum performance in terms of the evaluated physicomechanical and wear properties.

## 5. Conclusions

This study optimized the physicomechanical and sliding wear properties of wood waste and rice husk/wood waste (0, 2.5, 5, 7.5 and 10 wt.%) reinforced PLA biocomposites. The attributes in the selection procedure were physical (density, water absorption), mechanical (tensile, impact, compression and flexural) and sliding wear test results. The density of PLA-based biocomposites grew as the rice husk increased, while it decreased as the wood waste increased. With more rice husk and/or wood waste content, the strength of the PLA biocomposites (tensile, impact, compression and flexural) declined, whereas the modulus (tensile, compression and flexural) and water absorption increased. The wear of the PLA biocomposites was lowered when the rice husk/wood waste percentage was reduced. The biocomposites with 2.5 wt.% wood waste and 5 wt.% wood waste/rice husk added had the lowest wear, which rose when more filler was added. Since no PLA biocomposite satisfied all of the performance attributes, the hybrid MABAC–CRITIC approach was utilized to determine the preference of the biocomposite alternatives. The CRITIC technique was used to determine the weight of the selected attributes. By applying MABAC, the preference order of PLA biocomposites was obtained, and the biocomposite alternative of 7.5 wt.% wood waste exhibited the optimum properties. The application demonstrated that the MABAC technique, enhanced by CRITIC, is an effective decision-making tool for choosing biocomposite alternatives when the selected parameters are composition dependent, and they show no discernible pattern in physicomechanical and wear performances.

## Figures and Tables

**Table 1:** Ingredients and composition variation.

Ingredients	Composition (wt.%) of Biocomposite Alternatives								
	p-1	p-2	p-3	p-4	p-5	p-6	p-7	p-8	p-9
PLA	100	97.5	95	92.5	90	97.5	95	92.5	90
Rice husk	0	1.25	2.5	3.75	5	0	0	0	0
Wood waste	0	1.25	2.5	3.75	5	2.5	5	7.5	10

**Table 2:** The test conditions and implications of selected performance attributes.

Attribute	Test Condition	Performance Implication
q-1: Tensile strength (MPa)	EN ISO 527	Higher-the-better
q-2: Compressive strength (MPa)	EN ISO 604	Higher-the-better
q-3: Impact strength (kJ/m^2^)	EN ISO 179	Higher-the-better
q-4: Flexural strength (MPa)	EN ISO 178	Higher-the-better
q-5: Tensile modulus (GPa)	EN ISO 527	Higher-the-better
q-6: Compressive modulus (GPa)	EN ISO 604	Higher-the-better
q-7: Flexural modulus (GPa)	EN ISO 178	Higher-the-better
q-8: Density (g/cm^3^)	Archimedes’ principle	Lower-the-better
q-9: Water absorption (%)	ASTM D570-98	Lower-the-better
q-10: Wear (g)	Load = 50 N, sliding distance = 2.5 km, sliding velocity = 3 m/s	Lower-the-better

**Table 3:** Experimental data of the alternatives.

	Attributes										
		q-1: Tensile Strength (MPa)	q-2: Compressive Strength (MPa)	q-3: Impact Strength (kJ/m^2^)	q-4: Flexural Strength (MPa)	q-5: Tensile Modulus (GPa)	q-6: Compressive Modulus (GPa)	q-7: Flexural Modulus (GPa)	q-8: Density (g/cm^3^)	q-9: Water Absorption (%)	q-10: Wear (g)
Biocomposite alternatives	p-1	57.96 ± 0.27	105.67 ± 1.06	15.25 ± 1.66	99.53 ± 0.21	2.56 ± 0.04	2.71 ± 0.11	3.43 ± 0.02	1.240 ± 0.032	0.36 ± 0.015	0.1652 ± 0.004
	p-2	51.13 ± 0.97	101.40 ± 1.95	11.42 ± 0.88	99.67 ± 0.61	2.77 ± 0.05	3.25 ± 0.17	3.58 ± 0.02	1.263 ± 0.008	0.78 ± 0.026	0.1142 ± 0.002
	p-3	51.19 ± 1.44	100.84 ± 1.58	11.25 ± 1.32	99.33 ± 0.87	2.79 ± 0.04	3.36 ± 0.13	3.69 ± 0.07	1.266 ± 0.012	1.16 ± 0.012	0.1014 ± 0.002
	p-4	50.06 ± 0.31	102.12 ± 3.27	8.75 ± 1.50	97.84 ± 0.52	2.89 ± 0.06	3.45 ± 0.13	3.92 ± 0.03	1.272 ± 0.010	1.44 ± 0.021	0.1644 ± 0.003
	p-5	50.23 ± 0.51	101.86 ± 1.63	9.63 ± 1.05	97.36 ± 1.44	3.02 ± 0.05	3.58 ± 0.11	4.03 ± 0.03	1.277 ± 0.010	1.74 ± 0.032	0.2047 ± 0.003
	p-6	53.01 ± 0.62	99.44 ± 2.42	12.31 ± 2.25	100.20 ± 0.67	2.66 ± 0.04	2.94 ± 0.06	3.51 ± 0.01	1.225 ± 0.030	0.94 ± 0.021	0.1176 ± 0.003
	p-7	51.87 ± 0.54	100.86 ± 2.44	10.44 ± 0.52	100.43 ± 0.59	2.78 ± 0.04	3.36 ± 0.09	3.74 ± 0.03	1.211 ± 0.022	1.56 ± 0.035	0.1646 ± 0.005
	p-8	51.52 ± 1.31	100.68 ± 1.72	9.38 ± 1.79	99.96 ± 1.08	2.94 ± 0.04	3.43 ± 0.08	3.90 ± 0.04	1.198 ± 0.020	1.90 ± 0.040	0.2017 ± 0.005
	p-9	50.90 ± 0.41	99.98 ± 2.07	9.63 ± 1.80	99.01 ± 0.66	2.97 ± 0.04	3.46 ± 0.27	4.03 ± 0.02	1.183 ± 0.028	1.92 ± 0.035	0.2618 ± 0.006

**Table 4:** Normalized matrix.

		Attributes									
		q-1: Tensile Strength	q-2: Compressive Strength	q-3: Impact Strength	q-4: Flexural Strength	q-5: Tensile Modulus	q-6: Compressive Modulus	q-7: Flexural Modulus	q-8: Density	q-9: Water Absorption	q-10: Wear
Biocomposite alternatives	p-1	1.0000	1.0000	1.0000	0.7030	0.0000	0.0000	0.0000	0.3936	1.0000	0.6022
	p-2	0.1354	0.3146	0.4031	0.7591	0.4565	0.6207	0.2500	0.1489	0.7308	0.9202
	p-3	0.1430	0.2247	0.3876	0.6271	0.5000	0.7471	0.4333	0.1170	0.4872	1.0000
	p-4	0.0000	0.4302	0.0000	0.1320	0.7174	0.8506	0.8167	0.0532	0.3077	0.6072
	p-5	0.0215	0.3884	0.1240	0.0000	1.0000	1.0000	1.0000	0.0000	0.1154	0.3560
	p-6	0.3722	0.0000	0.5442	0.9241	0.2174	0.2644	0.1333	0.5532	0.6282	0.8990
	p-7	0.2291	0.2279	0.2543	1.0000	0.4783	0.7471	0.5167	0.7021	0.2308	0.6060
	p-8	0.1848	0.1990	0.0899	0.8449	0.8261	0.8276	0.7833	0.8404	0.0128	0.3747
	p-9	0.1063	0.0867	0.1287	0.5314	0.8913	0.8621	1.0000	1.0000	0.0000	0.0000

**Table 5:** Results of CRITIC method.

	q-1: Tensile Strength	q-2: Compressive Strength	q-3: Impact Strength	q-4: Flexural Strength	q-5: Tensile Modulus	q-6: Compressive Modulus	q-7: Flexural Modulus	q-8: Density	q-9: Water Absorption	q-10: Wear
$\chi_{j}$	2.5713	2.5996	2.6361	3.0507	3.6803	3.643	4.1872	3.5011	3.0887	3.1114
$\varpi_{j}$	0.0802	0.0810	0.0822	0.0951	0.1148	0.1136	0.1306	0.1092	0.0963	0.0970

**Table 6:** Weighted normalized matrix.

		Attributes									
		q-1: Tensile Strength	q-2: Compressive Strength	q-3: Impact Strength	q-4: Flexural Strength	q-5: Tensile Modulus	q-6: Compressive Modulus	q-7: Flexural Modulus	q-8: Density	q-9: Water Absorption	q-10: Wear
Biocomposite alternatives	p-1	0.1604	0.1620	0.1644	0.1620	0.1148	0.1136	0.1306	0.1522	0.1926	0.1554
	p-2	0.0911	0.1065	0.1153	0.1673	0.1672	0.1841	0.1633	0.1255	0.1667	0.1863
	p-3	0.0917	0.0992	0.1141	0.1547	0.1722	0.1985	0.1872	0.1220	0.1432	0.1940
	p-4	0.0802	0.1158	0.0822	0.1077	0.1972	0.2102	0.2373	0.1150	0.1259	0.1559
	p-5	0.0819	0.1125	0.0924	0.0951	0.2296	0.2272	0.2612	0.1092	0.1074	0.1315
	p-6	0.1100	0.0810	0.1269	0.1830	0.1398	0.1436	0.1480	0.1696	0.1568	0.1842
	p-7	0.0986	0.0995	0.1031	0.1902	0.1697	0.1985	0.1981	0.1859	0.1185	0.1558
	p-8	0.0950	0.0971	0.0896	0.1754	0.2096	0.2076	0.2329	0.2010	0.0975	0.1333
	p-9	0.0887	0.0880	0.0928	0.1456	0.2171	0.2115	0.2612	0.2184	0.0963	0.0970

**Table 7:** The border approximation area (${Β}_{j}$) matrix.

Attributes									
q-1: Tensile Strength	q-2: Compressive Strength	q-3: Impact Strength	q-4: Flexural Strength	q-5: Tensile Modulus	q-6: Compressive Modulus	q-7: Flexural Modulus	q-8: Density	q-9: Water Absorption	q-10: Wear
0.0976	0.1049	0.1067	0.1499	0.1759	0.1845	0.1967	0.1509	0.1303	0.1518

**Table 8:** The distance ($D_{ij}$) matrix.

		Attributes									
		q-1: Tensile Strength	q-2: Compressive Strength	q-3: Impact Strength	q-4: Flexural Strength	q-5: Tensile Modulus	q-6: Compressive Modulus	q-7: Flexural Modulus	q-8: Density	q-9: Water Absorption	q-10: Wear
Biocomposite alternatives	p-1	0.0628	0.0571	0.0577	0.0121	−0.0611	−0.0709	−0.0661	0.0013	0.0623	0.0036
	p-2	−0.0065	0.0016	0.0086	0.0174	−0.0087	−0.0004	−0.0334	−0.0254	0.0364	0.0345
	p-3	−0.0059	−0.0057	0.0074	0.0048	−0.0037	0.0140	−0.0095	−0.0289	0.0129	0.0422
	p-4	−0.0174	0.0109	−0.0245	−0.0422	0.0213	0.0257	0.0406	−0.0359	−0.0044	0.0041
	p-5	−0.0157	0.0076	−0.0143	−0.0548	0.0537	0.0427	0.0645	−0.0417	−0.0229	−0.0203
	p-6	0.0124	−0.0239	0.0202	0.0331	−0.0361	−0.0409	−0.0487	0.0187	0.0265	0.0324
	p-7	0.0010	−0.0054	−0.0036	0.0403	−0.0062	0.0140	0.0014	0.0350	−0.0118	0.0040
	p-8	−0.0026	−0.0078	−0.0171	0.0255	0.0337	0.0231	0.0362	0.0501	−0.0328	−0.0185
	p-9	−0.0089	−0.0169	−0.0139	−0.0043	0.0412	0.0270	0.0645	0.0675	−0.0340	−0.0548

## Data Availability

The data are available from the first author (T.S.) upon reasonable request.
